# *In-vivo* time course of organ uptake and blood-brain-barrier permeation of poly(L-lactide) and poly(perfluorodecyl acrylate) nanoparticles with different surface properties in unharmed and brain-traumatized rats

**DOI:** 10.3389/fneur.2023.994877

**Published:** 2023-02-06

**Authors:** Patrick Bechinger, Lucas Serrano Sponton, Verena Grützner, Anna Musyanovych, Daniel Jussen, Harald Krenzlin, Daniela Eldahaby, Nicole Riede, Oliver Kempski, Florian Ringel, Beat Alessandri

**Affiliations:** ^1^Department of Neurosurgery, Johannes Gutenberg University Medical Centre, Mainz, Germany; ^2^Department of Anesthesiology, Helios Dr. Horst Schmidt Clinic, Wiesbaden, Germany; ^3^Department of Neurosurgery, Sana Clinic Offenbach, Offenbach, Germany; ^4^Fraunhofer Institute for Microengineering and Microsystems, Mainz, Germany; ^5^Department of Neurosurgery, Johann Wolfgang Goethe University Frankfurt am Main, Frankfurt, Germany; ^6^San Paolo Medical School, Department of Health Sciences, Università degli Studi di Milano, Milan, Italy

**Keywords:** nanoparticles, controlled cortical impact, blood–brain barrier, traumatic brain injury, inflammation

## Abstract

**Background:**

Traumatic brain injury (TBI) has a dramatic impact on mortality and quality of life and the development of effective treatment strategies is of great socio-economic relevance. A growing interest exists in using polymeric nanoparticles (NPs) as carriers across the blood-brain barrier (BBB) for potentially effective drugs in TBI. However, the effect of NP material and type of surfactant on their distribution within organs, the amount of the administrated dose that reaches the brain parenchyma in areas with intact and opened BBB after trauma, and a possible elicited inflammatory response are still to be clarified.

**Methods:**

The organ distribution, BBB permeation and eventual inflammatory activation of polysorbate-80 (Tw80) and sodiumdodecylsulfate (SDS) stabilized poly(L-lactide) (PLLA) and poly(perfluorodecyl acrylate) (PFDL) nanoparticles were evaluated in rats after intravenous administration. The NP uptake into the brain was assessed under intact conditions and after controlled cortical impact (CCI).

**Results:**

A significantly higher NP uptake at 4 and 24 h after injection was observed in the liver and spleen, followed by the brain and kidney, with minimal concentrations in the lungs and heart for all NPs. A significant increase of NP uptake at 4 and 24 h after CCI was observed within the traumatized hemisphere, especially in the perilesional area, but NPs were still found in areas away from the injury site and the contralateral hemisphere. NPs were internalized in brain capillary endothelial cells, neurons, astrocytes, and microglia. Immunohistochemical staining against GFAP, Iba1, TNFα, and IL1β demonstrated no glial activation or neuroinflammatory changes.

**Conclusions:**

Tw80 and SDS coated biodegradable PLLA and non-biodegradable PFDL NPs reach the brain parenchyma with and without compromised BBB by TBI, even though a high amount of NPs are retained in the liver and spleen. No inflammatory reaction is elicited by these NPs within 24 h after injection. Thus, these NPs could be considered as potentially effective carriers or markers of newly developed drugs with low or even no BBB permeation.

## Introduction

Traumatic brain injury (TBI) is the major cause of death and disability in young adulthood and of considerable socio-economic relevance worldwide (1). Despite intensive translational research in the last decades, scarce advances in outcome improvement have been achieved up to the present (2, 3). Surely many factors associated with the very complex pathophysiology of TBI may have contributed to this lack of success (4, 5). However, as for many other neurological diseases, the presence of the blood-brain-barrier (BBB) constitutes a major limiting factor (6). The high efficiency of this barrier, required to maintain the homeostasis of the neural tissue and its microenvironment, as well as to protect it against exogenous noxa, makes the BBB an insurmountable obstacle for numerous pharmaceuticals to penetrate the brain parenchyma, thus preventing sufficient drug concentrations reaching the central nervous system (CNS). As a consequence, highly promising agents to treat CNS conditions often fail to show the expected successful outcomes (7–9). Even though it is well-known that TBI induces BBB disruptions, previous studies have clearly demonstrated that during the evolution of secondary trauma-associated injury mechanisms, BBB dysfunction with increased permeability occurs in phases, alternating with periods in which the BBB permeability remains intact (10–12). Therefore, it is probable that only drugs with the capability to overcome the BBB and act in both open and closed states of the BBB may provide effective results in the treatment of TBI.

An innovative and promising strategy to overcome the BBB obstacle has become the use of nanoparticles (NPs) specifically functionalized to cross the barrier and act as drug carriers into the CNS by introducing modifications of their physicochemical surface properties *via* adsorption or covalent linkage with surface-active substances (8, 13, 14). Although some of these surfactants have received increasing attention in recent years, others have been sparely studied. Several reports have shown that NPs coated with the non-ionic polysorbate80 (Tween-80^®^) can be absorbed into brain endothelial cells *via* apically located receptors according to the principle of a Trojan horse (15–20). In the case of other surfactants, such as the anionic surfactant sodium dodecyl sulfate (SDS), its capability to penetrate the BBB due to amphiphilic interactions with major membrane components has been less studied (18, 21).

However, demonstrating the ability of certain functionalized NPs to penetrate the BBB *in vitro* does not necessarily implicate its effectiveness to achieve this goal during *in vivo* conditions, given that NP bioavailability within the brain parenchyma is further influenced by serum clearance mechanisms and deposition in other organs (22–24). Furthermore, even if NPs as a means of transport for active substances offer numerous advantages, they harbor the risk of potential toxicity and induction of adverse inflammatory reaction, a phenomenon observed with NPs based on inorganic material (25–27). Even though biodegradable NPs are considered to possess a safer profile, their toxicity and immunogenicity are still to be further clarified (28, 29).

In this context, in the present study we aimed to comparatively evaluate the organ distribution, BBB penetration, brain bioavailability and possible induction of inflammatory responses driven by Tween-80 (Tw80) and SDS coated biodegradable poly(L-lactide) (PLLA) and non-biodegradable poly(perfluorodecyl acrylate) (PFDL) NPs after systemic administration in a rodent model. We further assessed the distribution of these NPs in the traumatized brain along regions with intact and disrupted BBB, in order to infer the potential application of NPs as carriers of neuroprotective agents for the treatment of TBI.

## Materials and methods

### Subjects

A total of 95 male Sprague-Dawley rats (Charles river, Sulzfeld, DE) weighing 250 ± 50 g were used. The animals were kept at a regular day/night cycle of 12/12 h, constant humidity of 50 ± 5%, a room temperature of 22 ± 2°C, and access to food and water *ad libitum*. After the operation, the animals were kept individually until euthanasia. All experiments described here had been approved by the Rhineland-Palatinate State Veterinary Office under file number 23 1770-G15-1-085. Animal husbandry and experimentation were in keeping with the standards stipulated by the European Commission Directive 2010/63/EU and the “Principles of Laboratory Animal Care” (NIH publication No. 86-23, revised 1985). During the entire period, all efforts had been made to minimize any potential suffering of the animals and the number of animals required.

### Experimental plan

For the preliminary trial to determine the fluorescence detectability of NPs in the brain on microscopic sections, 15 animals were used with intracerebroventricular (icv) NP administration (*N* = 3 for each tested NP and 3 sham animals without injected NPs nor CCI). The aim of this pretrial was to rule out methodological issues resulting in lack of florescence in the following experiments after intravenous administration. Subsequently, 80 animals were used for intravenous (iv) NP application, which were again randomly subdivided on two different subsets, (I) without (*N* = 40) and (II) with (*N* = 40) controlled cortical impact (CCI). Both subsets were further divided into two survival times (4 and 24 h) for the four different assessed NPs (PLLA-SDS, PLLA-Tw, PFDL-SDS, and PFDL-Tw), resulting in equal groups of 5 subjects each. For intraventricular and intravenous administration, NP dispersions were diluted with PBS (Dulbecco's Phosphate Buffered Saline 1X, Sigma-Aldrich; St. Louis, USA) depending on their corresponding solid content. An NP dispersion with a dose of 1,000 μg/kg body weight was prepared for intraventricular injection and 10,000 μg/kg body weight for intravenous administration. The animals were euthanized at their assigned survival time and the brain and peripheral organs were removed for immunohistochemical processing. Sham-operated animals with no NP injected nor CCI were used as the control group for immunohistochemical analysis of inflammatory markers.

### Preparation and characterization of PFDL-SDS, PFDL-Tw, PLLA-SDS and PLLA-Tw NPs

The synthesis and characterization of NPs was carried out at the Fraunhofer Institute for Microengineering and Microsystems (Fraunhofer IMM) in Mainz. These were provided as part of the cooperation within a joint project with the German Federal Ministry of Education and Research (FKZ 13N13258). Synthesis was carried out following previous preparation procedures, using the miniemulsion process (30, 31). SDS was chosen, as it is known as a very effective surfactant for the production of small and monodisperse polymer particles and previous intracellular uptake studies using different cell types showed a good cell compatibility of SDS-stabilized NPs after proper purification (30, 31).

The poly(perfluorodecyl acrylate) particles (PFDL) were synthesized by free radical miniemulsion polymerization of perfluorodecyl acrylate with the oil-soluble azo initiator in a direct oil-in-water system. The fluorescent dye N-(2,6-diisopropylphenyl)-perylene-3,4-dicarboximide (PMI), which fluoresces in the green region of the light spectrum when excited (absorption maximum 479 nm), was used as a marker for the fluorescence measurements. The organic phase consisted of 1g 1H,1H,2H,2H-perfluorodecyl acrylate, 50 mg of osmotic reagent hexadecane, 1 mg PMI, 25 mg initiator 2,2′-azobis(2-methylbutyronitril) (V59) and 1 g chloroform. The organic phase was mixed with the aqueous phase consisting of 144 mg SDS dissolved in 24 g demineralized water. The obtained PFDL-SDS NPs were washed by centrifugation from the residual surfactant before characterization. PFDL-Tw NPs stabilized with non-ionic surfactant were obtained by exchanging SDS with Tw80 through multiple centrifugation of PFDL-SDS NPs and redispersion in 1% Tw80 aqueous solution.

The poly(L-lactide) NPs (PLLA) were produced from preformed polymer using a combination of miniemulsion and solvent evaporation technology. For this purpose, 300 mg poly(L-lactide) and 0.30 mg of fluorescent dye PMI were first dissolved in 10 g chloroform and a miniemulsion was produced together with the 0.3% SDS solution as an aqueous phase by ultrasonic homogenization as described for PFDL NPs. In a second step, the chloroform was evaporated by heating at 40°C, which resulted in precipitation of PLLA inside the droplets and thus encapsulation of the PMI. The particles were stabilized with SDS as a surfactant, or alternatively by multiple centrifugations and redispersion with Tw80 as described for PFDL-Tw NPs.

After purification, all NPs were characterized in terms of particle size, size distribution and zeta potential. The average size and size distribution of NP was determined by dynamic light scattering (DLS) using a Nanoflex DLS (Microtrac Europe GmbH, Germany) at 23°C operating at a scattering angle of 180°. The zeta potential was determined with a Malvern Zetasizer Nano Z in 1 mM KCl buffer solution. The obtained data are following: PFDL-SDS (particle diameter 197 ± 21 nm, polydispersity index 0.52, zeta potential −56 mV); PFDL-Tw (particle diameter 201 ± 26 nm, polydispersity index 0.53, zeta potential −32 mV); PLLA-SDS (particle diameter 121 ± 23 nm, polydispersity index 0.12, zeta potential −45 mV); and PLLA-Tw (particle diameter 156 ± 27 nm, polydispersity index 0.22, zeta potential −22 mV). NP samples were prepared and aliquoted into light-protected Eppendorf reaction vessels until final use.

### Intraventricular NP application

Animals were firstly given an anesthetic induction using 100% isoflurane inhalation (Forene^®^, AbbVie, Wiesbaden, Germany), after which chloral hydrate (Pharmacy of the Johannes Gutenberg University, Mainz, Germany) 36 mg/ml was intraperitoneally injected in an initial dose of 1 ml/100 g. To maintain anesthesia, further intraperitoneal injections of chloral hydrate were given whenever necessary at half of the initial dose. Animals were placed in prone position on a heating mat and a body temperature of 37 ± 0.5°C. was maintained by means of feedback *via* a rectal temperature probe. The animals were then fixed in a stereotactic frame with a fixation clamp on the maxillary incisors and with two ear bars inserted into the external acoustic meatus on both sides. Hair covering the surgical field was shaved and ointment (Corneregel^®^, Dr. Mann Pharma, Berlin, Germany) was applied to both eyes to avoid corneal dryness. After disinfection with octenidindihydrochlorid and phenoxyethanol (Octenisept^®^, Schülke&Mayr, Norderstedt, Germany), a sagittal skin incision of ~20 mm was made along the midline of the skull. The scalp was mobilized and the periosteum detached from the top of the skull with a dissector. This was followed by disinfection and hemostasis with 3% hydrogen peroxide (H_2_O_2_). Under microscopic visualization, a trephination of the skull was performed using a high-speed drill in the area of the right lateral ventricle under continuous cooling with 0.9% saline solution. The stereotactic coordinates used for this, based on Bregma and Lambda as anatomical landmarks, were AP −0.9 mm, ML −1.6 mm, DV 3.5 mm (32). Injection into the right lateral ventricle was carried out using a Hamilton syringe with a capacity of 25 μl (Hamilton Robotics, Reno, USA). A total volume of 10 μl NP sample (100 ug NPs per 100 g body weight) was slowly administered over a period of 2 min. Care was taken to leave the injection cannula *in situ* 2 min before and 5 min after the injection in order to prevent the NP sample from accidentally escaping from the puncture channel. After removing the injection cannula, the drill hole was closed with Histoacryl^®^ tissue adhesive (B. Braun, Melsungen, Germany) and the skin was sutured. The fixation of the stereotactic frame was released, animals were transferred to cages and placed on a warming mat until they were fully awake. The postoperative analgesia consisted of tramadol (Ratiopharm, Ulm, Germany) at a dose of 1 mg/ml in the drinking water.

### Intravenous NP injection

After being anesthetized in an identical way as described above, the animals were placed prone on a heating mat keeping body temperature at 37 ± 0.5°C by means of feedback *via* a rectal temperature probe. For vasodilation, the tail was preheated for 5 min using an infrared heat lamp, followed by a tourniquet. After antiseptic disinfection, the lateral tail vein was punctured about 3 cm distal to the root with an indwelling venous catheter of size 26G, and the return of blood was monitored to check the position. The tourniquet was then released and the NP sample (1,000 ug NPs per 100 g body weight) was slowly administered intravenously. After completing the administration, the indwelling venous catheter was removed and direct pressure was applied to the puncture site until hemostasis occurred. Finally, the animals were transferred to cages and placed on a warming mat, and monitored until they were fully awake.

### Controlled cortical impact (CCI)

Animals were anesthetized, placed in prone position on a heating mat, and fixed on the stereotactic frame in an identical fashion as described above (see intraventricular injection). After disinfection, a longitudinal skin incision of ~20 mm was made along the midline of the skull. The scalp was mobilized and the periosteum was detached from the skull with a dissector. This was followed by disinfection with 3% hydrogen peroxide. A right parietal craniotomy with a diameter of ~7 mm was carried out under microscopic visualization with a high-speed drill. The craniotomy boundaries were the coronary suture rostrally and the sagittal suture medially. Great care was taken not to injure the dura and only animals with an intact dura mater were used for the experiment.

CCI was performed using a pneumatically-driven impact device (produced by L. Kopacz, Mainz University Medical Center), with a concave tip 6 mm in diameter. The system, consisting of an electronically controlled bolt, accelerated with compressed air, is a modification of the device used by Dixon et al. and has long been established in our research group (33, 34). The injury parameters were: 5 m/s impact velocity, 2 mm injury depth, and 200 ms dwell time. The impact was applied perpendicularly to the brain surface. The CCI device was then removed, the autologous bone flap was repositioned and fixed with adhesive and the wound was closed. The intravenous application of the NPs took place 15 min after CCI by lateral tail vein punction.

After the experiment, animals were transferred to individual cages, placed on a 37°C warming mat, and monitored until they were fully awake. Postoperative analgesia consisted again of tramadol drops at a dose of 1 mg/ml in the drinking water.

### Staining and histological analysis

For further histological work-up, animals were euthanized after their stipulated survival time (4, 24h). For this purpose, the animals were again anesthetized as previously described and transcardiac perfusion was carried out with buffered saline 0.9%. Brains were firstly extracted, followed by heart, lung, liver, spleen, and kidneys. Organs were shock-frozen in isopentane (Carl Roth, Karlsruhe, Germany) and dry ice for 3 min. All organs were separately wrapped in aluminum foil to protect them from light and stored in a freezer at −80°C until further use.

The frozen 12-μm-thick sections of the brain and peripheral organs were made at −18°C. Brain sections of intravenously injected animals were at Bregma level 0.00 mm, as well as −0.84, −2.04, and −3.00 mm following the rat's brain stereotaxic coordinates atlas as a reference (35). For animals injected intraventricularly, sections were made throughout the injection area.

Nuclear staining with 4′,6-diamidine-2-phenylindole (DAPI) was then carried out. For this, the sections were first dried at room temperature for 15 min, after which they were wetted with a drop of mounting medium each and immediately covered with a coverslip. Until microscopic evaluation on the following day, they were stored in a refrigerator at 4°C, protected from light.

To evaluate the colocalization of neurons and NPs, immunofluorescence staining against neuronal nuclei (NeuN) was performed. The slides with the frozen sections were first air-dried at room temperature for 15 min, followed by fixation with 4% paraformaldehyde (PFA), also for 15 min. They were washed in three steps with PBS for 5 min and then incubated for 1 h in normal donkey serum, diluted in 0.7% PBST (1,000 ml PBS + 3 ml Triton^®^ X 100, Merck Millipore; Burlington, USA) and 0.5% BSA (Bovine Serum Albumin, Sigma-Aldrich; St. Louis, USA), in order to block unspecific binding sites. The sections were then covered with 100 μl of the diluted primary antibody (Mouse Anti-NeuN, Merck Millipore; Burlington, USA) at a dilution of 1: 100 and incubated in humidity chambers at 4°C overnight. The primary antibody was diluted in PBST and 1% BSA. The next day, three washing steps were carried out in PBS for 5 min each. The sections were then incubated for 2 h with the respective secondary antibody. For this purpose, the sections were again wetted with 100 μl of the diluted secondary antibody. The incubation took place in staining chambers at room temperature. The antibody solution was again diluted with PBST and 1% BSA. After washing three times in PBS for 5 min each time, the sections were covered with DAPI mounting medium and cover glasses. Protected from light, these were stored in the refrigerator at 4°C until their microscopic evaluation.

Further immunofluorescence staining against the glial fibrillary acidic protein (GFAP) to display activated astrocytes, ionized calcium-binding adapter molecule 1 (Iba1) displaying the microglia, as well as CD31 targeting brain capillary endothelial cells (BCEC) were carried out analogously to the staining against NeuN described above. For these cases, Mouse Anti-GFAP (BD Pharmingen; San Diego, USA) in a dilution of 1: 100, Mouse Anti-Iba1 (Santa Cruz Biotechnology; Dallas, USA) in a dilution of 1:200, and Mouse Anti-CD 31 (Abcam; Cambridge, UK) in a dilution 1:10 were respectively used as primary antibodies.

In order to assess the eventual inflammatory reaction induced by the administered NPs, brain sections from animals without having undergone CCI were immunohistochemically stained on the basis of 3,3'-diaminobenzidine (DAB Peroxidase, HRP Substrate Kit SK 4100, Vector Laboratories, Burlingame, USA). After oxidation with H_2_O_2_, positive staining is obtained as a brown dye complex in light microscopy. For this purpose, sections were first dried for 10 min at room temperature and then fixed for 10 min using PFA 4%. The endogenous peroxidase activity was then blocked using methanol and hydrogen peroxide (100 ml methanol + 1 ml H_2_O_2_ + PBS). Methanol and H_2_O_2_ were then removed by rinsing once with 70% ethanol. This was followed by preincubation with 5% normal serum in PBST for 30 min. For this purpose, normal horse serum was used for the staining against GFAP and TNFα, and normal rabbit serum for the staining against Iba1 and IL1β (each from the Vectastain^®^, Elite^®^, Vector Laboratories; Burlingame, USA). The sections were then treated with the respective primary antibody against activated astrocytes (anti-GFAP, dilution 1: 100), microglial cells (anti-Iba1, dilution 1: 100), tumor necrosis factor α (Mouse Anti-TNFα, ab1793, Abcam, Cambridge, UK, dilution 1:50) and Interleukin 1β (Goat Anti-IL1β, ab9787, Abcam, Cambridge, UK, dilution 1:50), dissolved in 1% BSA and PBST and incubated in a humidity chamber moistened with tap water overnight at 4°C. In parallel, negative controls were carried out on each slide. After the sections were washed three times with PBST again, they were incubated with the respective biotin-conjugated secondary antibody (dilution 1: 100 in 1% BSA and PBST) for 30 min at room temperature. This was followed again by three washing steps with PBST for 5 min each, followed by incubation with the avidin-biotin complexes an enzymatic amplification system (ABC-Kit HRP, Vectastain^®^, Vector Laboratories, Burlingame, USA; used according to the manufacturer instruction). The sections were then washed three times with PBS for 5 min and stained using DAB (DAB Substrate Kit SK4100) for 2–5 min according to the manufacturer's instruction. After washing and dewatering with isopropyl alcohol, sections were placed in xylene. They were covered with a Eukitt medium (Orsatec, Bobingen, Germany) and cover glasses and protected from light until their microscopic evaluation.

### Definition of regions of interest

For the microscopic analysis of NP distribution, regions of interest (ROI) with a size of 363 × 273 μm were formed at 40 × magnification in each organ. Within the brain, the ROI in non-CCI animals was specifically allocated in the area of the right parietal cortex. For this, brain slices at the Bregma −3.00 mm were used. In animals undergoing CCI, ROI-1 corresponded to the lesioned area and ROI-2 was determined in the hippocampus, located ipsilateral but distant to the injured cortex. In these animals, a further mirrored ROI-1 was placed on the contralateral side to the trauma, at approximately the same distance from the midline in order to enable a comparison of the ipsi- and contralateral effects. For the analysis of inflammatory reaction, the ROIs were determined in the same way. When magnified 20 times, they had a size of 865 × 486 μm.

Stains were evaluated with digital fluorescence microscopy at 40× magnification. Excitation time and microscope's filter settings were left unchanged during the entire evaluation in order to objectify the results. The immunoreactive areas were determined using ImageJ 1.52n [(36), NIH, Bethesda, USA]. For this purpose, the image files were first converted into grayscale images. Then a threshold value was set, in such a way that the fluorescence signal of the NPs was optimally displayed. The threshold set was then transferred to all analyzed images. With the analysis function, the colored area was finally measured in relation to the total area of the ROI evaluated. The immunohistochemical stains were scanned and evaluated at a 20 × magnification. The sections of the respective ROIs were then defined in an image processing program (NDPView 2.5.19, Hamamatsu Photonics, Hamamatsu, Japan). The assessment of the immunoreactive area was performed again with ImageJ. Here the threshold was chosen in such a way that only the structures clearly stained by the respective coloring were visible. The measurement of the immunoreactive area was also carried out in the manner described above.

### Statistical analysis

All statistical analysis and graphic representations were carried out with SigmaPlot 12.5 for Windows (Systat Software, San Jose, USA). Parametric or non-parametric distribution of each variable was checked using the Shapiro-Wilk test. To compare the individual groups, a One-Way ANOVA analysis of variance was performed with normally distributed data and the results were tested for individual significant group differences using the Student–Newman–Keuls *post-hoc* test. If there was no normal distribution, the Kruskal–Wallis ANOVA on Ranks and, as a *post hoc* test, the Dunnett's test to compare several test groups against a control group, as well as the Student–Newman–Keuls test were used. To compare the ipsi- and contralateral ROIs, the cortex and hippocampus, as well as NP uptake at 4 and 24 h for each organ the paired *t*-test was used for normally distributed data and the Wilcoxon signed rank test for non-normally distributed data. Statistical significance was assumed for *p* < 0.05. The Holm–Sidak method was used to adjust for multiple testing. The respective level of significance is identified as follows: ^***^p < 0.001, ^**^p < 0.01, ^*^p < 0.05. All data are shown as mean ± standard error of the mean (SEM).

## Results

### Determination of NP fluorescence in brain slices after intraventricular application

**Figure 1:**
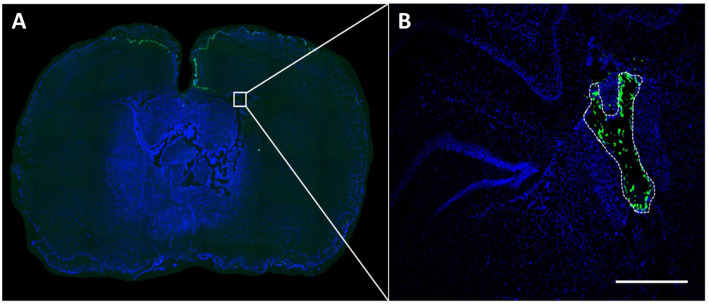
NP fluorescence after intraventricular application. A volume of 10 μl NP preparation (1,000 μg/kg body weight) was injected into the right lateral ventricle and detectability of NP fluorescence was assessed in brain sections 1 h after application. The green fluorescence signal (in this case corresponding to PLLA-SDS NPs) can be easily differentiated from the DAPI at bregma −0.92 **(A)**. Microscopic magnification shows NP distribution within the lateral ventricle **(B)**. White bar indicates 100 μm.

10 μl of each NP sample was intraventricularly injected into the right lateral ventricle of animals euthanized after 1 h. Histological processing confirmed NP fluorescence both intraventricularly and within the brain parenchyma for all NP types (Figure 1).

### Organ distribution of NPs after intravenous injection

**Figure 2:**
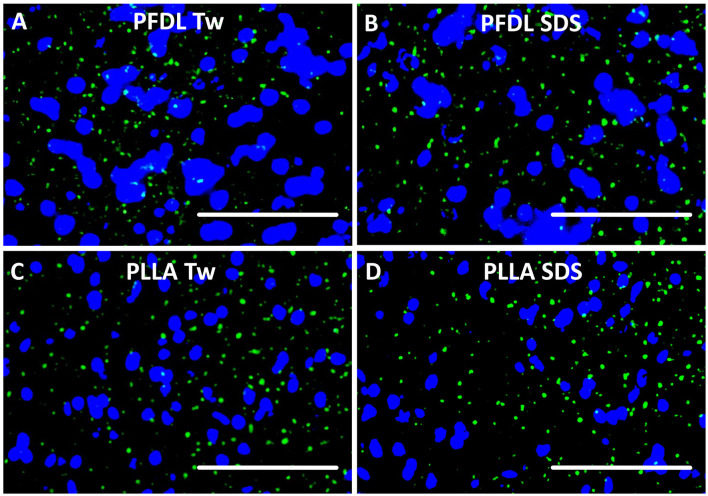
NP visualization after intravenous application. Each NP has been injected intravenously at a dose of 10,000 μg/kg body weight. Tissue sections were yielded 4 and 24 h after treatment and evaluated under fluorescence microscopy. In this example, green fluorescence of PFDL-Tw **(A)**, PFDL-SDS **(B)**, PLLA-Tw **(C)**, and PLLA-SDS **(D)** can be identified and differentiated from the DAPI in brain sections, taken from the right parietal cortex (Bregma −3.00 mm), 4 h after intravenous application. White bars indicate 50 μm.

The fluorescence of all NP types was detected on sections after intravenous administration (Figure 2).

**Figure 3:**
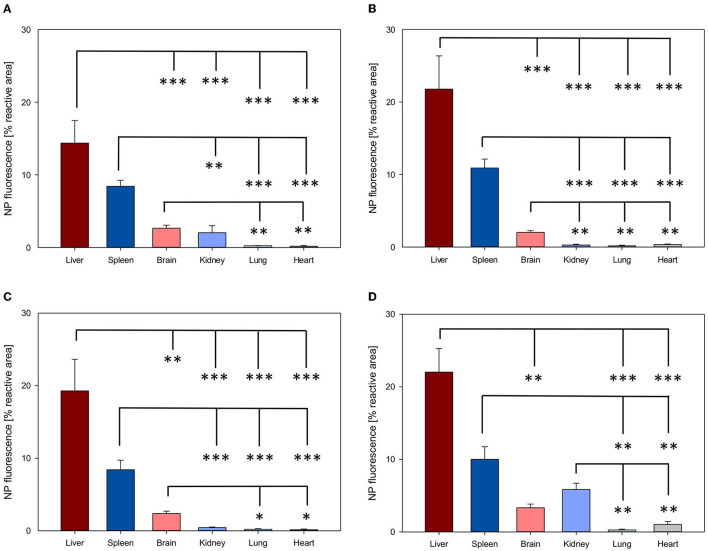
Organ distribution of NP uptake 4h after intravenous injection. The accumulation of PFDL-Tw **(A)**, PFDL-SDS **(B)**, PLLA-Tw **(C)**, and PLLA-SDS **(D)** nanoparticles in all organs was assessed as the percentage of reactive fluorescent area in relation to the total area of the ROI at 40 × magnification. Overall, one-way ANOVAs showed higher NP uptake in the liver and spleen, followed by a moderate uptake in the brain and the kidney and minimal in the lung and the heart. Bars express means ± SEM. ***p < 0.001, **p < 0.01, *p < 0.05.

**Figure 4:**
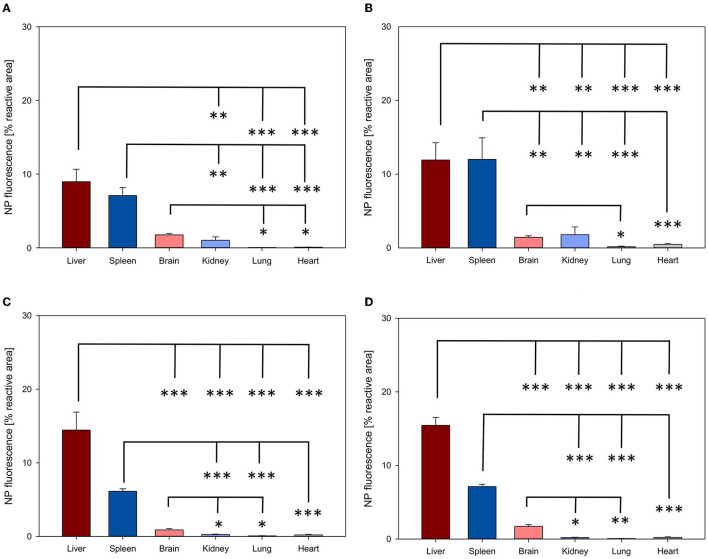
Organ distribution of NP uptake 24 h after intravenous injection. The accumulation of PFDL-Tw **(A)**, PFDL-SDS **(B)**, PLLA-Tw **(C)**, and PLLA-SDS **(D)** nanoparticles in all organs was assessed as the percentage of reactive fluorescent area in relation to the total area of the ROI at 40 × magnification. Overall, one-way ANOVAs showed higher NP uptake in the liver and spleen, followed by a moderate to low accumulation in the brain and the kidney and very low in the lung and the heart. Bars express means ± SEM. ***p < 0.001, **p < 0.01, *p < 0.05.

NP biodistribution at 4 and 24 h after i.v. administration was assessed in the liver, spleen, brain, kidney, lung, and heart and their signal was quantified as the percentage of the fluorescent area from the total area of the ROI. A similar organ distribution pattern after 4 and 24 h was evidenced for all four NPs assessed (Figures 3, 4).

Among all organs, a clear statistically significant higher NP uptake was found in the liver followed by the spleen. A relevant but less pronounced NP uptake was found in the brain, whereas NP accumulation in the heart and lungs was significantly low in all cases (all *p* < 0.001 vs. liver, Figures 3, 4). NP accumulation in the kidney 4 h after injection was more pronounced for PLLA-SDS followed by PFDL-Tw. In both cases, a comparable mean uptake into the brain was found. For the case of PFDL-SDS and PLLA-Tw, uptake in the kidney after 4 h was as low as that observed in the lungs and heart. At 24 h, both PFDL NPs were found in the kidney in similar concentrations as in the brain, whereas kidney accumulation of PLLA NPs was significantly reduced in comparison to the brain (both *p* < 0.05).

**Figure 5:**
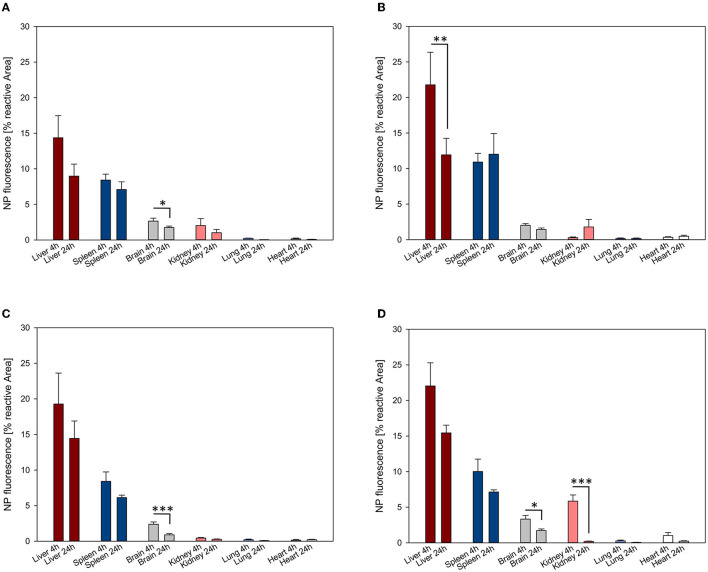
NP uptake kinetics assessed at 4 and 24 h in the liver, spleen, brain, kidney, lung and heart. The accumulation of PFDL-Tw **(A)**, PFDL-SDS **(B)**, PLLA-Tw **(C)**, and PLLA-SDS **(D)** nanoparticles was assessed as the percentage of reactive fluorescent area in relation to the total area of the ROI at 40 × magnification. Paired *t*-tests were used for comparing NP uptake at both study time points for each organ, showing in general a trend to decay in NP accumulation after 24 h. Bars express means ± SEM. ***p < 0.001, **p < 0.01, *p < 0.05.

An overall trend of reduction in NP accumulation was evidenced after 24 h in all organs, except for PFDL-SDS accumulation in the spleen and the kidney, for which a trend to higher accumulation was observed after 24 h (Figure 5).

A statistically significant reduction in NP accumulation after 24 h was demonstrated in the brain for all NPs except for PFDL-SDS (PFDL-Tw = *p* < 0.05, PLLA-Tw = *p* < 0.001, PLLA-SDS = *p* < 0.05), in the liver for PFDL-SDS (*p* < 0.01) and the kidney for PLLA-SDS (*p* < 0.001).

**Figure 6:**
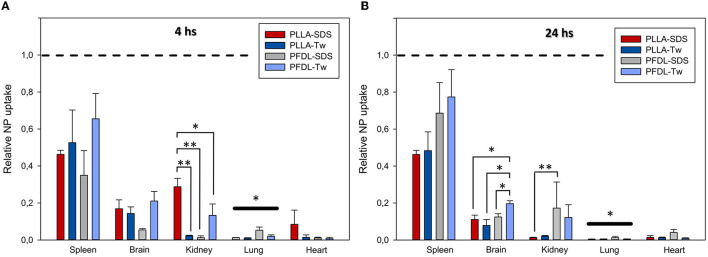
Comparison of the relative NP uptake in the spleen, brain, kidney, lung and heart at 4 **(A)** and 24 h **(B)** after intravenous injection. Given the differences in fluorescence dye intensity between NP types, the percentage of reactive fluorescent area values of each organ were normalized in relation to liver values (depicted at *y* = 1 with dotted lines) in order to enable a comparison between NP-types. The overall significantly lower relative NP uptake in the lung compared to other organs at both study points is depicted by bold significant level bars. Data were assessed using one-way ANOVA. Bars express means ± SEM. **p < 0.01, *p < 0.05.

Since the strength of fluorescent dye differed among NP types, data normalization was performed for direct comparative uptake assessment between NPs in different organs. Given that the highest NP uptake was observed in the liver, the mean values of the reactive areas of other organs were normalized to the values in the liver. The evaluation of the relative fluorescence showed only a statistically significant lower overall NP uptake in the lung compared to other organs at 4 and 24 h (both *p* < 0.05, Figure 6).

A trend toward increased uptake of the PFDL-Tw NPs was observed in the spleen and brain compared to the other NPs at both study time points. A separate analysis of relative NP uptake in each organ revealed only statistically significant differences in the brain at 24 h and the kidney at 4 and 24 h. At 4 h, relative PLLA-SDS uptake was significantly higher in the kidney than PLLA-Tw (*p* < 0.01), PFDL-SDS (*p* < 0.01), and PFDL-Tw (*p* < 0.05). After 24 h, the relative uptake of PLLA-SDS in the kidney was significantly reduced in comparison to PFDL-SDS (*p* < 0.01) and a trend to reduced PLLA-Tw uptake was also observed when compared to PFDL-SDS, although the difference remained below the significance level. No differences were observed in relative NP distribution in the brain at 4 h. However, a significantly higher accumulation of PFDL-Tw in comparison to the other 3 NP-types was evidenced at 24 h (all *p* < 0.05).

### NP uptake into the intact and injured brain

**Figure 7:**
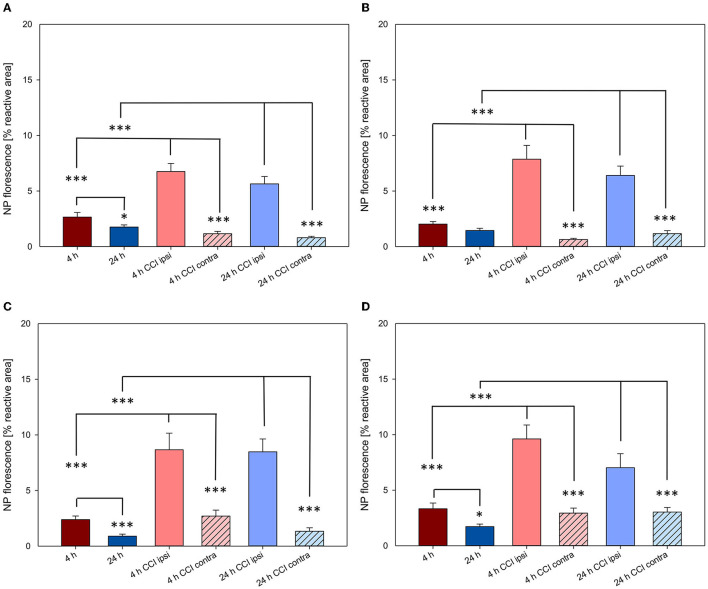
NP uptake in the brain with and without CCI at 4 and 24 h after intravenous application. All NPs showed a higher uptake in the injured hemisphere at both time points. The accumulation of PFDL-Tw **(A)**, PFDL-SDS **(B)**, PLLA-Tw **(C)**, and PLLA-SDS **(D)** nanoparticles in the brain was assessed as the percentage of reactive fluorescent area in relation to the total area of the ROI at 40 × magnification. One-way ANOVAs were used to compare NP uptake in brains without CCI (ROI within the right parietal cortex, Bregma −3.00 mm), with the lesioned area (ipsi CCI) and the mirrored ROI on the contralateral hemisphere (contra CCI) of traumatized brains. Bars express means ± SEM. ***p < 0.001, *p < 0.05.

As previously described, in animals with intact brain conditions and not having undergone CCI, brain accumulation of NPs decreased from 4 to 24 h, reaching significance for both PLLA NPs and PFDL-Tw (Figures 5, 7). After CCI-induced brain injury, there was a drastic significant, and consistent increase of NP uptake at 4 and 24 h within the traumatized hemisphere in comparison to the contralateral hemisphere and the brain of uninjured animals for all NP-types (all *p* < 0.001, see Figure 7).

**Figure 8:**
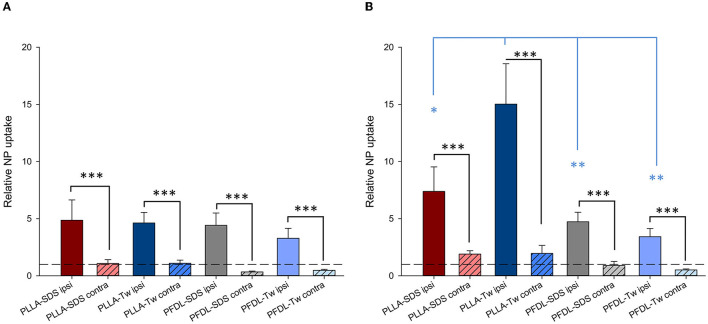
Comparison of the relative NP uptake in CCI injured brains between the ipsi- and contralateral hemisphere at 4 **(A)** and 24 h **(B)** after intravenous NP injection. Due to the differences in fluorescence intensity between NP types data were normalized as described in the method section. The dotted lines depicted at *y* = 1 represent the value from uninjured brain tissue. Paired *t*-tests were used for comparing ipsi- vs. contralateral ROIs in CCI injured animals (black asterisks), whereas one-way ANOVA was used for comparing the relative NP uptake within the injured area between different NP types (blue asterisks). Bars express means ± SEM. ***p < 0.001, **p < 0.01, *p < 0.05.

Again, in order to compare the accumulation of NPs in the brain between the different NP-types, normalization was performed, in this case to the mean uptake values of the respective experimental group without CCI at the same time point (for example, PLLA-SDS 4h CCI ipsilateral—or contralateral—hemisphere, normalized to PLLA-SDS 4h brain without CCI, Figure 8).

**Figure 9:**
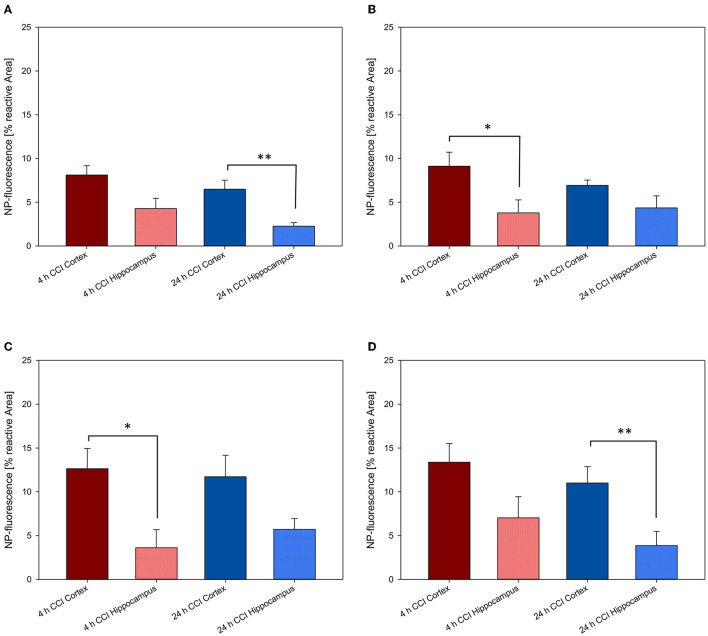
Comparison of NP uptake between the cortical and hippocampal area within the CCI-injured hemisphere at 4 and 24 h after intravenous injection. The accumulation of PFDL-Tw **(A)**, PFDL-SDS **(B)**, PLLA-Tw **(C)**, and PLLA-SDS **(D)** nanoparticles was assessed as the percentage of reactive fluorescent area in relation to the total area of the ROI at 40 × magnification. Paired *t*-tests showed a clear trend to higher NP uptake in the cortex compared to the hippocampus, located away from the CCI area. Bars express means ± SEM. **p < 0.01, *p < 0.05.

The significantly higher NP concentration in the CCI-injured hemisphere in comparison to the contralateral side was still evident for all NPs at both 4 and 24 h (all *p* < 0.001). No uptake differences were observed between NP-types at 4 h within the traumatized hemisphere. At 24 h, the relative uptake of PLLA-Tw was significantly higher than the other NP-types within the CCI-injured hemisphere (vs. PLLA-SDS = *p* < 0.05, vs. PFDL-SDS and PFDL-Tw = *p* < 0.01). To further assess differences in NP distribution within the injured hemisphere, NP accumulation in the perilesional area was compared to the uptake in the hippocampus, located away from the CCI-induced lesion. For this purpose, the mean values of ipsilateral ROI-1 (referred to as “cortex”) and the mean values of ROI-2 (the ipsilateral hippocampus) were compared (Figure 9).

A clear trend for higher NP uptake in the perilesional area compared to the hippocampus was observed for all NP-types at 4 and 24 h. This difference reached statistical significance at 4 h for PFDL-SDS and PLLA-Tw (both *p* < 0.05) and at 24 h for PFDL-Tw and PLLA-SDS (both *p* < 0.01).

### NP uptake in neurons, glial and brain capillary endothelial cells (BCEC) and assessment of inflammatory response

**Figure 10:**
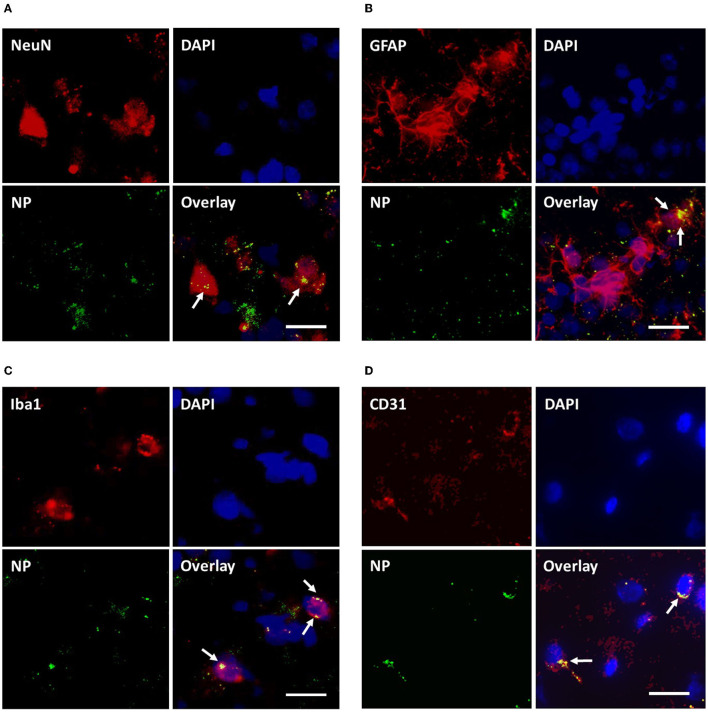
Assessment of cellular uptake of PLLA-SDS by fluorescence microscopy. NP internalization into neurons, astrocytes, microglia and brain capillary endothelial cells was qualitatively studied by assessing the co-localization of NP-green fluorescence with red-fluorescence of antibodies against NeuN **(A)**, GFAP **(B)**, Iba1 **(C)**, and CD31 **(D)** in red fluorescence. Co-localization was evidenced by overlaying yellow fluorescent areas (white arrows). White bars indicate 25 μm.

As depicted in Figure 10, qualitative microscopic evaluation demonstrated the co-localization of NPs in neurons, microglia, astrocytes, and BCEC at both 4 and 24 h after intravenous NP administration.

**Figure 11:**
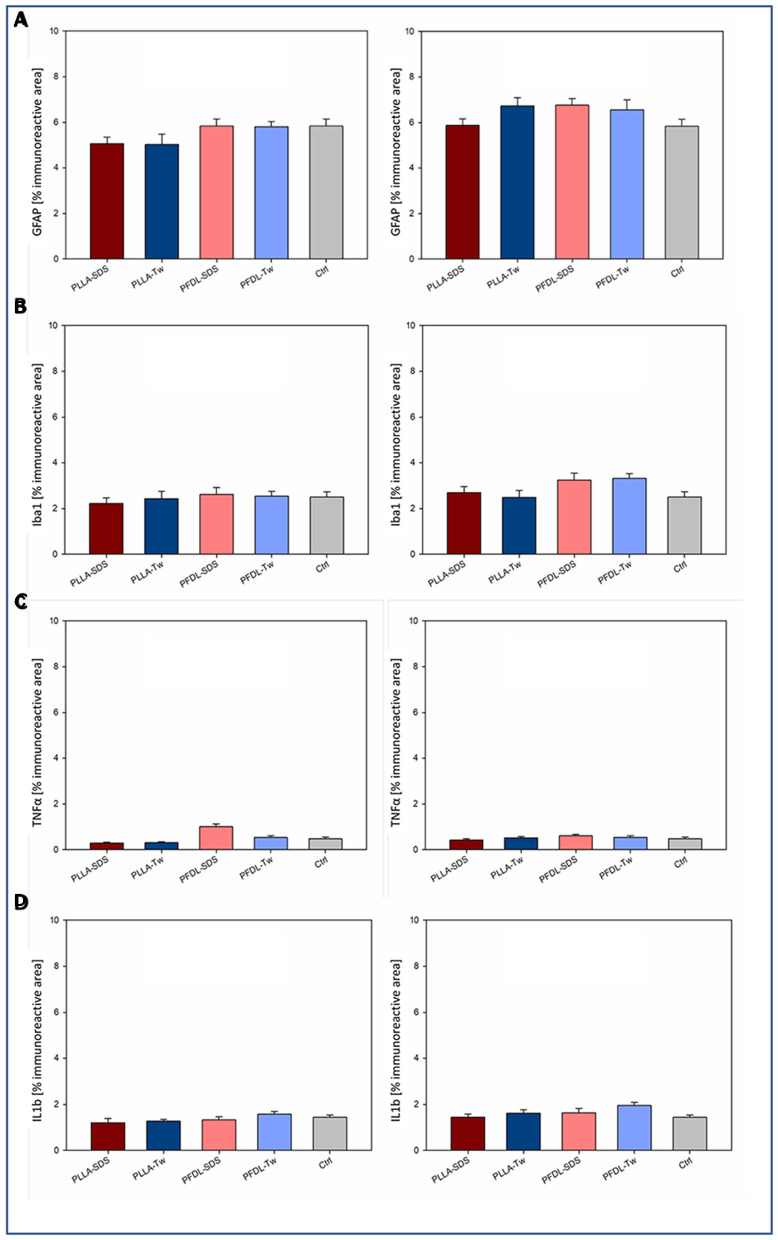
Assessment of a neuroinflammatory reaction by intravenously injected NPs. Immunohistochemical staining against two markers of glial activation: GFAP **(A)**, Iba1 **(B)**; and two proinflammatory cytokines: TNFα **(C)**, and IL1β **(D)** was compared between uninjured animals with and without NP injection at the survival timepoints of 4 and 24 h. One-way ANOVAs showed no statistically significant differences in immunoreactive areas between groups. Bars express means ± SEM.

Potential neuroinflammatory effects due to uptake of NPs into the brain were assessed through immunohistochemical staining against GFAP and Iba1 as markers of astrocyte and microglia activation. In addition, immunohistochemical staining against TNFα and IL1β was performed to detect possible increased expression of proinflammatory cytokines in neuronal tissue (Figure 11).

Immunohistochemical staining against GFAP showed no significant difference in immunoreactive areas between all NP-treated groups compared to the control, neither at 4 nor at 24 h after intravenous NP application (Figure 11A). Evaluation of immunohistochemical staining against Iba1 showed also no significant difference in immunoreactive areas compared to controls at both evaluation times (Figure 11B). Consistent with these results, no significant increase in TNFα expression was observed among the different groups after NP injection compared to the control (Figure 11C). A trend toward a slight increase of TNFα expression in the PFDL-SDS group 4 h after NP application remained below statistical significance and completely disappeared at 24 h. There was also no evidence of higher proinflammatory interleukin 1β expression in the NP-injected groups in comparison to the control (Figure 11D). The impression of a slightly higher IL1β expression at 4 and 24 h after PFDL-Tw application remained below statistical significance.

## Discussion

### Organ distribution of NPs

A high NP distribution in the liver and spleen have been reported on diverse polymeric NPs in contrast to a lower accumulation in the kidney and brain and a very low uptake in the lungs and heart (37, 38). Our data also confirm the overall higher NP accumulation in the examined organs described in many publications a few hours after injection and the decreasing NP uptake over time (39–42). A comparison of uptake between the four intravenously administered Tw80 and SDS coated biodegradable (PLLA) and non-biodegradable (PFDL) NPs used in our experiment revealed a similar overall organ distribution, although a higher uptake of PLLA-SDS than PLLA-Tw and PFDL NPs in the kidney 4 h after application and a preponderance of PFDL NP accumulation in the kidney and brain in comparison to both PLLA NPs assessed 24 h after injection was observed.

The size of the NPs used in this work did not markedly differ between NP-types (with a difference of only ~80 nm between slightly smaller PLLA-SDS and both PFDL NPs), so a clear difference in organ uptake between NP-types driven specifically by differential NP size was not expected. Although some evidence shows that larger NPs with a diameter of over 200 nm might be absorbed more quickly in the liver and spleen due to a capillary filter effect, other studies have demonstrated an inverse relationship between particle size and uptake in these organs (37, 43–47). A possible explanation for this phenomenon might be the fact that NP uptake depends more on the area of surface exposed than on NP volume (48). Some NPs with small diameters have significantly more extensive areas in relation to their mass, offering a higher potential for interaction with plasma proteins, which may lead to the formation of larger NP-protein complexes that can be easily retained in the liver and spleen (47). Moreover, the higher concentration of mononuclear phagocyte system (MPS) cells in the liver and spleen represents the major determinant for the higher NP accumulation in these organs, an event indirectly related to NP surface characteristics. The surface properties of NPs are known to determine the features of the protein corona (formed when the NP comes into contact with the bloodstream), which acts as opsonins for immune cells of the MPS (49). Scavenge of NPs by cells of the MPS resident in the liver and spleen explain the particularly highly extended accumulation of NPs in these organs, which is also supported by our results of NP distribution (50, 51). Consistent with this, studies assessing the uptake kinetics of poly(lactic-co-glycolic acid) (PLGA) NPs have demonstrated their predominant internalization in CD68-positive Kupffer cells of the liver (52).

The rate of renal clearance of NPs is largely determined by NP size. Very small NPs with a molecular weight below 5,000 Da or a particle diameter smaller than 10 nm are rapidly eliminated renally as they can pass through the glomerular filter unhindered (44, 53, 54). In the case of larger NPs, such as the ones used in this study (ranging from 121 ± 23 to 201 ± 26 nm), the high first-passage NP retention in the liver and spleen, in addition to a higher difficulty in crossing the glomerular pores may be responsible for a predominant biliar excretion with reduced renal uptake and clearance (55). Nevertheless, renal accumulation of larger biodegradable PLGA-NPs (between 140 and 214 nm) has been demonstrated to decrease after 24 h (39, 42). We suggest that the architectural changes in the biodegradable PLLA-NPs (which start to occur after a few hours) may have contributed to the faster glomerular filtration and a trend to more accelerated decay in renal accumulation when compared to stable non-biodegradable PFDL-NPs (56, 57).

As seen in this and previous works, the retention of NPs in peripheral organs constitutes a major obstacle to achieving a substantial concentration of NPs in the brain. It has been demonstrated that NP linkage with polyethyleneglycol chains (PEGylation) is effective to reduce serum protein adsorption, therefore minimizing opsonization and MPS retention in the liver and prolonging NP residence in the blood circulation (50, 58). However, the effectiveness shown by PEGylated NPs to avoid opsonization has been linked to a reduced adsorption of apolipoproteins, which, on the other hand, play a relevant role in the mechanisms of BBB penetration (19, 50, 59). Tw80 has been demonstrated to improve brain concentration of ropinirole-hydrochloride-loaded chitosan NPs, reducing their accumulation in the liver, spleen and kidney and decreasing by ~40% the accumulation of [^14^C]-poly(butyl cyanoacrylate) NPs in MRS organs 1 h after injection, in addition to increasing uptake into the brain 2-fold in a similar manner (60, 61). Nevertheless, in the study of Ambruosi et al. the absolute brain uptake of polysorbate-80-coated NPs remained below 1% of the total amount of NPs injected, measured at different time points (1, 6, 24 h, 3 days, etc), and far below the uptake into the liver (60). Our experiments showed that Tw80 coated NPs were still taken up very strongly in the liver and spleen in absolute measures. There were also no significant advantages observed for any NP composites (PLLA, PFDL) nor in their coated surfactants (SDS and Tw80) used in this study in reducing hepatic and splenial steal, suggesting a similar effectiveness of both surfactants in reducing the interaction with opsonins and the macrophagic system. In the light of these results, even though coating NPs with surface-active substances may reduce their retention in peripheral organs and increase their accumulation within the brain tissue, the development of more effective strategies to improve brain uptake rate should be a matter of further research.

### NP uptake into the brain

Several organic polymeric, as well as inorganic NPs have been demonstrated to possess the property of overcoming the BBB and entering the brain parenchyma after systemic administration (62, 63). An advantage of polymeric NPs over the other NP types is their biocompatibility and, in many cases, biodegradable properties, thus decreasing the risk of harmful effects due to longer-term deposition into the brain (50). Previous works have shown highly promising results when using polymeric NPs coated with Tw80 and poloxamer 188 as carriers for neuroprotectant agents in the treatment of CNS conditions, such as in animal models of Parkinsonian syndrome, scopolamine-induced amnesia or TBI (64, 65). The mechanisms for crossing the BBB are partially linked to surfactant molecules coated on the selected NPs. As mentioned before, in the case of Tw80 coated NPs, the adsorption of apolipoproteins ApoE and ApoA from the blood plasma as part of the protein corona formation has been shown to stimulate the interaction with low-density lipoprotein receptors LRP1 and LRP2 on brain endothelial cells, leading to receptor-mediated NP transcytosis (59, 66). In the case of SDS, its capability to disrupt the BBB due to amphipilic interactions with major membrane components, as well as by interacting with specific biochemical intracellular pathways of brain endothelial cells has been also demonstrated in previous works (21, 67, 68). Although mechanisms of internalization of SDS coated NPs through the BBB have been less studied, it has been observed that the protein corona is mainly composed of various apolipoproteins, suggesting that SDS-functionalized NPs could be also taken up into the brain tissue *via* an interaction with LDL receptors (69–71).

Besides corona components and receptor interactions, NP size represents a further relevant factor for transiting the BBB. Indeed, the inverse relationship between particle size and uptake of NPs within the brain parenchyma has been demonstrated in numerous studies (38, 72–74). It has been pointed out that the ideal diameter to overcome the BBB seems to be in the range of 10 to 200 nm, a criterion that fits with all NPs used in the present study (44). As for peripheral organs, no differential size-dependent NP brain uptake was expected, given the minor differences in size of the NPs used. Consistent with this, no statistically significant differences in brain accumulation were evidenced at 4 h between NPs. However, a higher brain concentration of PFDL NPs with both stabilizers was observed at 24 h, when compared to PLLA NPs. We speculate that other factors unrelated to the minimal differences in NP size and more likely associated to the degradability of PLLA NPs may explain their faster disappearance in brain slices 24 h after injection.

Regarding the temporal kinetics of NP uptake into brain tissue, our results demonstrated an overall trend to higher uptake in the brain after 4 h, followed by a decrease 24 h after NP injection, which reached statistical significance for all NPs except PFDL-SDS. These findings are consistent with previous reports which illustrated a higher NP concentration in the brain after 2 or 4 h with a further decrease toward 24 h in inorganic, as well as in polymeric PLGA-NPs with different functionalizations (41, 60, 75, 76). We observed an acceptable NP uptake measured on brain sections, but the values were far below those from the liver or spleen. The early clearance of NPs from the bloodstream by peripheral organs is mainly responsible for a diminished amount of NPs in brain tissue. Despite their potential to cross the BBB, usually even <5% of the total amount of injected NPs reaches the brain parenchyma (60, 77, 78). Although a specific determination of the percentage of NPs that entered the brain cannot be extracted from our data, the percentage of the reactive area shown by each NP in brain sections was barely below 5% at 4 h and continued decreasing at 24 h.

As soon as NPs have entered the brain parenchyma, they can present neurotoxic or neuroinflammatory potentials, as seen for inorganic NPs. Administration of metallic NPs made of copper, aluminum and silver lead to BBB disturbances, neuron destruction, glial activation and heat shock protein up-regulation within 24 h (79, 80). Silver NPs may trigger the release of proinflammatory cytokines, most notably TNFα and IL1β and titanium dioxide NPs induce glial and neuroinflammatory activation (81–84). The findings on the wide-ranging neuroinflammatory potential of inorganic NPs led to further development of biocompatible and biodegradable NPs made of biological polymers. Biodegradable polymeric NPs have been shown in various *in vitro* studies to have no deleterious effects on astrocytes (85). However, the passage of NP-protein corona complexes into the CNS could theoretically lead to inflammatory effects driven by protein components that normally do not overcome the BBB. Prior studies have shown no negative effects on brain cell viability for PLGA and PLLA NPs (69, 70, 86), although dose-dependent cytotoxicity of PLGA NPs on retinal microvascular endothelial cells has been documented (87). In an *in-vitro* study, we detected no harmful influence on cell viability of astrocytes and cerebral microvascular endothelial cells by the same PLLA and PFDL NPs used in this study (69, 70). Consistent with these *in-vitro* findings, even though the internalization of PLLA and PFDL NPs in astrocytes and microglia was evident *in-vivo*, no significant activation of these cells was observed within 24 h after intravenous application. Moreover, we found no evidence of increased expression of TNFα and IL1β triggered by the NPs used. In line with our results, Tw80 functionalized chitosan NPs induced no *in-vivo* astrocyte activation (88). Likewise, *in-vitro* studies show that biocompatible PBCA NPs stabilized with SDS, Tw80 or having other functionalization do not induce increased expression of proinflammatory cytokines (89, 90).

Taken together, the NPs used in this study induced no elevated positive staining and did not differ in their elicited inflammatory reaction under both conditions suggesting no elevated inflammatory response against the applied materials. This is an important finding for the use of such particles within a living body as drug carrier or marker.

### NP uptake in the brain after CCI

A significant higher NP uptake was evidenced in the traumatized compared to the intact brain, a phenomenon that is clearly related to BBB hyperpermeability, which is known to reach a peak 1 h after CCI, remains elevated for 4–6 h, and decreases to a level barely above normal at 24 h (10, 91–94). To a lesser extent, NP uptake was still observed in the hemisphere contralateral to the CCI, where the BBB is known to remain intact (94). The transfer of NPs across a compromised BBB and their distribution in the brain after TBI has only been addressed in a very limited number of *in-vivo* studies. In a cryolesion TBI model, it was observed that after injection of PLGA-NPs of different sizes, the brain uptake was especially high in the injured area for NPs averaging a diameter of 100 nm (37). The maximum concentration in the lesion area was reached 1 h after injection and decreased significantly over the course of 24 h. The use of brain-derived neurotrophic factor-(BDNF)-loaded PLGA-NPs coated with poloxamer 188 has been demonstrated to significantly increase BDNF concentrations in the ipsi- and contralateral hemisphere after focal TBI in C57Bl/6 mice, improving the neuroprotective effect of BDNF and neurocognitive outcome in injured animals (64). After CCI, the temporal and spatial distribution of non-biodegradable carboxylated polystyrene NPs of sizes between 20 and 500 nm demonstrated a high NP uptake in the injured area 1 h after trauma, followed by a significant reduction within 24 h. NPs were injected at defined times after CCI (0, 2, 5, 12, and 23 h) and the uptake in brain tissue was always analyzed after a circulation period of 1 h (72). This made it possible to establish a more direct relationship to the time course of the BBB disturbance, but not to the accumulation over time. In the present work, no significant difference was detectable in the time course between the uptake in the lesion area 4 and 24 h after CCI. The procedure we have chosen reflects the entire residence time of the NPs in the circulation and is thus also subject to possible influences such as redistribution and degradation as would occur during the treatment of patients. In general, brain accumulation of NPs transported across an intact BBB drops within 24 h after administration. The BBB breakdown due to CCI induced a prolonged NP uptake and thus possible treatment efficacy.

In addition, a highly significant difference in NP accumulation between the BBB-injured and BBB-intact areas in CCI subjects was observed. We found that the relative NP uptake on the lesion side was on average 6-fold higher compared to the contralateral side. This is in accordance with the findings of other groups, which have demonstrated a significant difference in NP uptake between the ipsi- and contralateral side following CCI injury and hemispheric differences of 5.2-fold at 6 h after penetrating trauma (72, 95). Furthermore, regional differences in NP uptake between the CCI affected area and intact regions within the same traumatized hemisphere were found in our experiments. This replicates previous findings showing that NP accumulation remains the highest close to the injury site (72). The higher NP uptake in the injured CCI area induced by BBB disruption seems to be a local phenomenon and does not affect the regular NP uptake into normal brain tissue (96). Thus, locally enhanced drug delivery by NPs is focused at the site of cellular distress which could improve treatment efficacy and at the same time reduce possible adverse effects of the drug and its carrier. Since the BBB may fluctuate between open and closed states after TBI (10–12), effective therapeutic strategies for TBI should be designed to guarantee considerable brain uptake of pharmacological agents during both states of intact and disrupted BBB. In this context, all four assessed NPs demonstrated acceptable brain penetration under intact as well as compromised BBB condition after CCI. Overall, the amount of NPs 4 and 24 h after CCI was independent of NP-type and stabilizer by SDS or Tw80. The higher accumulation 24 h after trauma of PLLA-Tw NPs in the CCI area, when normalized to the low mean values of this NP in the brain of non-CCI subjects (probably driven by NP degradation) supports the evidence that CCI-induced BBB breakdown leads not only to a higher, but also to a prolonged retention of NPs in the lesioned area.

Besides the capability to enter the brain parenchyma another step for successful pharmacological treatment of brain diseases such as focal brain injury is the differential internalization of NPs into BCEC, neurons, astrocytes, or microglia. At an intact BBB, the BCEC has to be initially overcome by transcytosis. Several NPs including ApoE-modified and Tw80 functionalized PBCA NPs have demonstrated their capability to enter mice and rat BCEC in *in-vitro* co-cultures, a fact that represents the first condition required for the process of transcytosis across the BBB (97, 98). The further *in-vivo* analysis demonstrated the internalization of Tw80 functionalized PBCA NPs into the BCEC and the brain, strongly suggesting transcytosis through the BBB in the rat (98). *In-vitro* evidence of time- and concentration-dependent uptake of PLLA and PFDL NPs also comes from incubating porcine astrocytes and microvascular endothelial cells with 150 μl/ml NPs revealing a maximal uptake within 20 h and no further increase at 48 h (70).

*In-vivo*, all NPs used were taken up not only in BCEC, but also in astrocytes, neurons, and microglia. Internalization of NPs by astrocytes is relevant in the context of the main role played by these cells in protective and deleterious pathophysiological mechanisms, such as during secondary lesion development in TBI or neurodegenerative disorders (99–103). Consistent with our findings, some polymeric NPs have been proven to be useful as carriers for antiretroviral therapy in combination with antioxidant and anti-inflammatory neuroprotectants into astrocytes, reducing the oxidative stress and neuroinflammation caused by human neurotropic immunodeficiency virus (104). Also, biodegradable PLGA NPs have even been used for gene delivery into astrocytes aiming to compensate for astrocytic dysfunction linked to neurodegeneration (103).

The uptake of NPs within neurons is also relevant, since the neuronal membrane is known to constitute a further major barrier for drug delivery, when a sufficient intraneuronal drug concentration is required (101, 105). Methods used to overcome this barrier relying on liposomes or other charged lipid formulations present limited complex stability in blood and often high toxicity over time (101, 106). Viral-based vectors have shown limited efficacy while raising several concerns in terms of safety (107). By contrast, the use of polymeric NPs may represent an effective alternative to overcome these limitations, given their stability and biocompatibility. Polybutylcyanoacrylate and PEGylated polyester NPs have been demonstrated not only to be able to cross the BBB (15, 105, 108), but also to be internalized into the neurons (101). It has been suggested that the mechanism of internalization across the BBB mediated by Apo-E and LDL receptors may also permit NP uptake into the neurons and it is possible that the same mechanism may be responsible for neuronal uptake observed for our NPs with their Apo-E containing corona (17, 59, 101).

Our experiments also demonstrated NP uptake in microglia. Similar to neurons and astrocytes microglia are involved in pathophysiological mechanisms linked to neurological conditions (109). As an example, *in-vitro* and *in-vivo* experiments have demonstrated the internalization of drug-loaded polymeric poly(methyl methacrylate) NPs in activated microglia and drug release into the cytosol of these cells, thus being a promising potential tool able to counteract secondary inflammatory events in spinal cord injury (102). As potential drug carriers, biodegradable PLGA NPs, iron-oxide NPs, carbon nanotubes as well as quantum dots have been shown to be internalized in microglial cells (110–113). Since LDL receptors are also found on microglia (114), we assume that the tested surfactant-coated NPs with their corona composition could have entered these cells *via* LDL-receptor interactions.

Although all NPs used in our experiments showed internalization into neuronal, glial, and endothelial cells, whether this uptake was homogeneous for all cell types cannot be inferred from our qualitative data. It has been demonstrated that NP internalization into different CNS cells is often heterogeneous, even though the mechanisms determining the selectivity for individual cell types are not fully understood (113, 115, 116). Despite further experiments aiming to obtain a quantitative NP distribution are still desirable, the present results demonstrate that the studied NPs can in principle be internalized into different CNS cells to a relevant degree. This suggests that they might be potentially useful when drug delivery is intended to target glial cells and neurons simultaneously. A higher selectivity for specific cell types could be achieved by additional specific functionalization of NPs.

## Conclusions

A major sink for the used PLLA and PFDL NPs has been the liver and spleen which reduces the availability of these NPs in other organs such as the brain. Despite the loss of NPs in peripheral organs, SDS and Tw80 coated polymeric PLLA and PFDL NPs show a considerable uptake into the brain parenchyma by being able to cross the intact BBB without specific differences between nanoparticles within the first 24 h after application. They also seem not to induce an inflammatory reaction which is a prerequisite for potential applications within living organisms. The disruption of the BBB by a CCI injury increased the available amount of NPs by 6-fold close to the traumatized region. If loaded with pharmaceuticals, the used PLLA and PFDL particles with SDS and Tw80 residuals could carry their content (drug or marker) to the brain region with the highest therapeutical needs and by dramatically reducing systemic adverse effects that conventional formulations may cause. Apart from the injury site, the NP concentration decreases gradually. Moreover, all NPs are taken up by neurons, astrocytes, microglial and microvascular endothelial cells, but with a high prevalence at the site of BBB breakdown and injured tissue. Modifications of these NPs for a cell-type-specific uptake would further improve the efficacy of pharmacological treatment following TBI.

## Summary

In the present study, systemically applied polymeric nanoparticles were shown to distribute mainly to the liver and spleen within 24 h after intravenous administration. To a lesser extent, uptake into the brain also occurred. In general, there were no statistically significant differences in brain uptake between the individual nanoparticles, besides a moderate higher accumulation of non-biodegradable NPs at 24 h. After crossing the blood-brain barrier, the NPs were internalized in microvascular endothelial cells, microglia, and astrocytes, as well as in neurons. Disruption of the blood-brain barrier, induced by controlled cortical impact, led to a highly significant increase in NP accumulation in the brain area directly adjacent to the lesion. The investigated NPs did not trigger activation of glial cells or increased release of proinflammatory cytokines in brain tissue in a period of 24 h after intravenous application. Taken together with the findings obtained in previous works, it can be summarized that BBB disruption in the context of TBI leads to a significant increase in NP uptake in the damaged brain area, thus opening a window for the targeted transport of neuroprotective agents for the treatment of secondary brain injury.

## Data availability statement

The raw data supporting the conclusions of this article will be made available by the authors, without undue reservation.

## Ethics statement

The animal study was reviewed and approved by Landesuntersuchungsamt Rheinland-Pfalz Referat 23 Mainzer Str. 112 56068 Koblenz Germany.

## Author contributions

PB, VG, and BA conceptualized this study. PB, LS, and DJ performed animal surgery. PB and LS performed the histological sections and analyzed the data. LS wrote the manuscript. AM contributed to the production of the nanoparticles used. PB, NR, and DE carried out histological staining. PB, LS, DE, and HK analyzed the images. BA, OK, and FR administrated the project. All authors reviewed the manuscript critically and approved for publication of the content.
